# Estimate of FDG Excretion by means of Compartmental Analysis and Ant Colony Optimization of Nuclear Medicine Data

**DOI:** 10.1155/2013/793142

**Published:** 2013-09-28

**Authors:** Sara Garbarino, Giacomo Caviglia, Massimo Brignone, Michela Massollo, Gianmario Sambuceti, Michele Piana

**Affiliations:** ^1^Dipartimento di Matematica, Università di Genova, Via Dodecaneso 35, 16146 Genova, Italy; ^2^CNR—SPIN, Via Dodecaneso 33, 16146 Genova, Italy; ^3^Dipartimento di Ingegneria Navale, Elettrica, Elettronica e delle Telecomunicazioni, Università di Genova, Via Opera Pia 11, 16145 Genova, Italy; ^4^IRCCS San Martino IST, Largo Rosanna Benzi 10, 16132 Genova, Italy; ^5^Dipartimento di Scienze della Salute, Università di Genova, Largo Rosanna Benzi 10, 16132 Genova, Italy

## Abstract

[^18^F]fluoro-2-deoxy-D-glucose (FDG) is one of the most utilized tracers for positron emission tomography (PET) applications in oncology. FDG-PET relies on higher glycolytic activity in tumors compared to normal structures as the basis of image contrast. As a glucose analog, FDG is transported into malignant cells which typically exhibit an increased radioactivity. However, different from glucose, FDG is not reabsorbed by the renal system and is excreted to the bladder. The present paper describes a novel computational method for the quantitative assessment of this excretion process. The method is based on a compartmental analysis of FDG-PET data in which the excretion process is explicitly accounted for by the bladder compartment and on the application of an ant colony optimization (ACO) algorithm for the determination of the tracer coefficients describing the FDG transport effectiveness. The validation of this approach is performed by means of both synthetic data and real measurements acquired by a PET device for small animals (micro-PET). Possible oncological applications of the results are discussed in the final section.

## 1. Introduction

Positron emission tomography (PET) [1] is an imaging technique capable of detecting picomolar quantities of a labeled tracer with temporal resolution of the order of seconds. FDG-PET [2–4] is a PET modality in which [^18^F]fluoro-2-deoxy-D-glucose (FDG) is used as a tracer to identify several kinds of tumors, since malignant cells typically exhibit an increased FDG uptake in PET scans. Although FDG is a glucose analog, there are several differences between the uptake and metabolism of FDG compared with normal glucose. Both molecules, for example, are transported into cells by the same proteins, and both are phosphorylated by hexokinase. However, FDG is trapped within the cell as a consequence of phosphorylation, and therefore it cannot be further metabolized; unlabeled glucose, on the contrary, is either stored as glycogen or rapidly metabolized. Further, at a renal level, unlike glucose, FDG is not reabsorbed by the renal tubule, and then it is excreted, which implies that there is radioactivity in the bladder. We point out that this last issue has a significant consequence as far as the effectiveness of the imaging procedure is concerned; in fact, the more radioactivity is in the bladder, the less amount of tracer is available for tumor identification.

A typical way to assess the FDG excretion in the urine is to compute the average clearance defined as the ratio between the (asymptotic) activity in the bladder and the time integral of the tracer concentration in blood [5]. In nuclear imaging, both these quantities can be computed by drawing regions of interest (ROIs) on the bladder and on the left ventricle and by computing the corresponding activities at different time points. However, clearance provides just a macroscopic description of the FDG metabolism with no information on the local tracer kinetics. From an experimental viewpoint, we notice that the measurement of the activity in these ROIs for human beings is made difficult (or even impossible) by the acquisition modalities typical of PET (simultaneous imaging of the left ventricle, the kidneys, and the bladder for several time intervals would require total body long acquisition time). In the present paper, we utilize a PET system for small animals (mice) to show that this local information on tracer kinetics can be inferred by applying a nonstandard compartmental analysis to nuclear data. We find, in particular, that a specific tracer coefficient determined by reducing the compartmental model is strongly correlated to average clearance and that the other computed coefficients provide a reliable local description of the effectiveness with which FDG is exchanged between the different physiological compartments.

**Figure 1:**
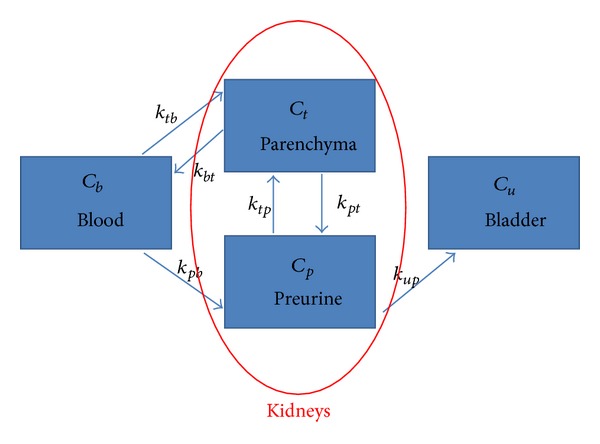
The compartmental model adopted in this paper.

The first novelty of our approach is in the kind of compartmental model adopted. Unlike the typical schemes for the study of renal physiology [6, 7], which are made of two functional compartments anatomically embedded in the kidneys, here we add a third compartment representing the pool where the excreted tracer is accumulated. Therefore, here we deal with the scheme represented in Figure 1.

- As in [6, 7], kidneys include two compartments. Differently from those papers, here we use the terms tissue or parenchyma (instead of plasma) and preurine (instead of tubules) for the two compartments. In particular, the reason of the choice of the term preurine is because in this compartment we encode two FDG functions: the one of a (possibly small) quantity of FDG that may in principle return to tissue and then to the plasma stream and the one of a bigger quantity of FDG that is captured by the tubular system and is excreted in the urine without reabsorption.
- A third compartment, the urine, is localized in the bladder and is characterized by one single input and no corresponding output.
- The time activity curve (TAC) describes the input of tracer in the system and is determined from regions of interest (ROIs) of the left ventricle drawn on FDG-PET maps at different time steps.
- Estimates of the tracer concentration for the bladder and for the two-compartment system made of parenchyma and preurine are obtained by means of ROIs including bladder and kidneys, respectively.
- Six exchange coefficients describe the efficiency of tracer transmission between the different compartments (further exchange coefficients, e.g., the ones describing the circulation for the bladder back to the kidneys, are set to zero for well-established physiological reasons).

From a mathematical viewpoint, the time-dependent concentrations of tracer in each compartment constitute the state variables; the time evolution of the state variables (the kinetics of the system) is modeled by a linear system of ordinary differential equations for the concentrations, expressing the conservation of tracer during flow between compartments; the (constant) coefficients describing the input/output rates of tracer for each compartment, called *exchange coefficients* or *rate constants* or *microparameters* [8], represent the unknowns to be estimated. Under the assumption that the exchange coefficients are given, we solve the direct problem of determining the explicit formal expressions of the tracer concentration in each compartment and classify the solutions in four families by means of spectral analysis arguments. Then, the inverse problem of determining the six unknown exchange coefficients is addressed by means of an “ad hoc” implementation of Ant Colony Optimization (ACO) [9, 10], which represents the second novelty of our computational approach to the assessment of FDG excretion. ACO is a statistical optimization algorithm inspired by evolutionary strategies that, in this application, minimizes the functional measuring the discrepancy between the experimental concentrations and the analytical forms provided by the solution of the forward problem. This functional is characterized by a notable number of local minima, and therefore for its minimization, biology-inspired strategies are certainly more effective than deterministic nonlinear optimization techniques [11]. Further, the gradient of this same functional has singularities (mainly in some specific directions), and therefore gradient-based schemes may be rather dangerous. 

The paper content is organized as follows. In Section 2, we describe the Cauchy problem modeling the direct problem and determine the exchange coefficients by means of statistical optimization.  Section 3 shows some applications to synthetic and real PET measurements. Our conclusions are offered in Section 4.

## 2. Materials and Methods

### 2.1. The Direct Problem

The state variables of the three-compartment model adopted in this paper are the tracer concentrations in the tissue (*C*_*t*_), in the preurine (*C*_*p*_), and in the urine contained in the bladder (*C*_*u*_) (all these concentrations are in Bq mL^−1^). Moreover, the kinetic process in the system is initialized by the TAC  *C*_*b*_, representing the tracer concentration in blood. The usual conditions for compartmental analysis hold; for example, tracer is uniformly distributed in each compartment at each instant [12], diffusive effects are neglected, and physiological processes are in a steady state. The six constant exchange coefficients (rate constants, in minutes^−1^) between compartments in contact are denoted as  *k*_*ab*_, where the suffixes  *a*  and  *b*  denote the target and source compartment, respectively. For example,  *k*_*up*_  is the rate constant of tracer carried “to” the bladder pool  *u*  “from” the preurine pool *p*. We assume  *k*_*ab*_ ≥ 0  for all cases.

Conservation of tracer exchanged between compartments leads to the following system of linear ordinary differential equations with constant coefficients:

(1) $$\begin{array}{c} {\dot{C}}_{t}=-\left(k_{bt}+k_{pt}\right)C_{t}+k_{tp}C_{p}+k_{tb}C_{b},\\ {\dot{C}}_{p}=k_{pt}C_{t}-\left(k_{tp}+k_{up}\right)C_{p}+k_{pb}C_{b},\\ {\dot{C}}_{u}=k_{up}C_{p}, \end{array}$$

with initial conditions *C*_*t*_(0) = *C*_*p*_(0) = *C*_*u*_(0) = 0, and where the dependence on time is implicit. The first two equations in (1) can be written in the compact form

(2) $$\begin{array}{c} \dot{C}=AC+KC_{b}, \end{array}$$

with initial condition  **C**(0) = 0. Here, in fact,

(3) $$\begin{array}{c} C∶=\left[\begin{array}{c} C_{t}\\ C_{p} \end{array}\right]; \end{array}$$

(4) $$\begin{array}{c} A=\left[\begin{array}{cc} -\left(k_{bt}+k_{pt}\right) & k_{tp}\\ k_{pt} & -\left(k_{tp}+k_{up}\right) \end{array}\right]=:\left[\begin{array}{cc} -a & b\\ c & -d \end{array}\right]; \end{array}$$

(5) $$\begin{array}{c} K:=\left[\begin{array}{c} k_{tb}\\ k_{pb} \end{array}\right]. \end{array}$$

The definition of  **A**  corresponds to

(6) $$\begin{array}{c} a∶=k_{bt}+k_{pt},\;\;c∶=k_{pt},\\ d∶=k_{tp}+k_{up},\;\;b∶=k_{tp} \end{array}$$

with inverse relations

(7) $$\begin{array}{c} k_{pt}=c,\;\;k_{bt}=a-c,\\ k_{tp}=b,\;\;k_{up}=d-b. \end{array}$$

In the first part of this subsection, we will show how the structure of matrix  **A**  is related to the explicit forms of tracer concentration in the different compartments. In the framework of compartmental analysis, this issue is crucial for the study of the inverse problem performed in the next sub-section.

Let us first assume that *c* ≠ 0 and *b* ≠ 0. Then, the solution of (2)–(7) is given by

(8) $$\begin{array}{c} C_{t}=c_{1}bE_{1}+c_{2}\;\left(d+\lambda_{2}\right)E_{2}, \end{array}$$

(9) $$\begin{array}{c} C_{p}=c_{1}\left(a+\lambda_{1}\right)E_{1}+c_{2}cE_{2}, \end{array}$$

(10) $$\begin{array}{c} E_{i}=\int_{0}^{t}e^{\lambda_{i}\;(t-\tau )}C_{b}\left(\tau \right)d\tau =e^{\lambda_{i}t}*C_{b},\;i=1,2, \end{array}$$

where ∗ denotes the convolution operator, the constants *c*_1_ and *c*_2_ are defined as

(11) $$\begin{array}{c} c_{1}=\frac{-ck_{tb}+\left(d+\lambda_{2}\right)k_{pb}}{\left(a+\lambda_{1}\right)\left(d+\lambda_{2}\right)-bc},\\ c_{2}=\frac{\left(a+\lambda_{1}\right)k_{tb}-bk_{pb}}{\left(a+\lambda_{1}\right)\left(d+\lambda_{2}\right)-bc}, \end{array}$$

and  *λ*_1_, *λ*_2_  are the eigenvalues of the matrix  **A**  with explicit values

(12) λ1=−(a+d)+(a+d)2−4(ad−bc)2,λ2=−(a+d)−(a+d)2−4(ad−bc)2.

We observe that both  *λ*_1_  and  *λ*_2_  are negative. The concentration  *C*_*u*_  is evaluated by inserting (9) in the third equation of (1), which leads to

(13) Cu(t)kup=∫0tCp(σ)dσ=∫0t[c1(a+λ1)E1(σ)+c2cE2(σ)]dσ.

We utilize the identity

(14) ∫0t∫0σC(τ)  ew  (σ−τ)dτdσ=1w  ∫0tC(σ)ew  (t−σ)dσ−1w∫0tC(τ)dτ,

where  *C*  is any continuous function and  *w*  is a real parameter and find

(15) Cu(t)kup=c1(a+λ1)λ1E1+c2cλ2E2−[c1(a+λ1)λ1+c2cλ2]∫0tCb(τ)dτ.

We now suppose that  *b* = 0  and  *c* ≠ 0  in the definition of the matrix  **A**; that is,

(16) $$\begin{array}{c} A=\left[\begin{array}{cc} -a & 0\\ c & -d \end{array}\right]. \end{array}$$

In this case, we set  *λ*_1_ = −*a*  and  *λ*_2_ = −*d*. This corresponds to vanishing denominators in (11). Integration of the linear system leads to

(17) $$\begin{array}{c} C_{t}=\chi_{1}\left(\lambda_{1}-\lambda_{2}\right)E_{1},\\ C_{p}=c\;\chi_{1}E_{1}+\chi_{2}E_{2}, \end{array}$$

where  *E*_1_  and  *E*_2_  are convolutions defined as in (10) and

(18) $$\begin{array}{c} \chi_{1}=\frac{k_{tb}}{\lambda_{1}-\lambda_{2}},\;\;\chi_{2}=k_{pb}-\frac{ck_{tb}}{\lambda_{1}-\lambda_{2}}. \end{array}$$

Moreover, integrating the third equation in (1) leads to

(19) $$\begin{array}{c} \frac{C_{u}}{k_{up}}=\frac{c\chi_{1}}{\lambda_{1}}E_{1,b}+\frac{\chi_{2}}{\lambda_{2}}E_{2,b}-\left[\frac{c\chi_{1}}{\lambda_{1}}+\frac{\chi_{2}}{\lambda_{1}}\right]\int_{0}^{t}C_{b}\left(\tau \right)\;d\tau .\; \end{array}$$

We then consider the case  *b* ≠ 0,  *c* = 0; that is,

(20) $$\begin{array}{c} A=\left[\begin{array}{cc} -a & b\\ 0 & -d \end{array}\right]. \end{array}$$

Again we have  *λ*_1_ = −*a*  and  *λ*_2_ = −*d*. The solution of the Cauchy problem for this upper triangular system is

(21) $$\begin{array}{c} C_{t}=\sigma_{1}E_{1}+b\sigma_{2}E_{2},\\ C_{p}=\left(\lambda_{2}-\lambda_{1}\right)\sigma_{2}E_{2}, \end{array}$$

with

(22) $$\begin{array}{c} \sigma_{1}=k_{tb}-\frac{bk_{pb}}{\lambda_{2}-\lambda_{1}},\;\;\sigma_{2}=\frac{k_{pb}}{\lambda_{2}-\lambda_{1}} \end{array}$$

and  *E*_1_,  *E*_2_  defined as in (10).

Finally, we consider the diagonal case  *b* = 0,  *c* = 0; that is,

(23) $$\begin{array}{c} A=\left[\begin{array}{cc} -a & 0\\ 0 & -d \end{array}\right]. \end{array}$$

Then, it is straightforward to obtain

(24) $$\begin{array}{c} C_{t}=k_{tb}E_{1},\;\;C_{p}=k_{pb}E_{2},\\ C_{u}=-k_{pb}E_{2}+k_{pb}\int_{0}^{t}C_{b}\left(\tau \right)d\tau . \end{array}$$

### 2.2. Solution of the Inverse Problem

The model equations obtained in the previous section describe the time behavior of the tracer concentration in the three compartments of the renal system, given the TAC for tracer concentration in blood and the transmission coefficients. Given such equations, compartmental analysis requires the determination of the tracer coefficients by

1. utilizing measurements of the tracer concentrations provided by nuclear imaging;
2. applying an optimization scheme for the solution of the inverse problem.

In nuclear imaging experiments, the reconstructed images can provide information on the tracer concentration in the kidneys and in the bladder as well as in the input arterial blood as measured in the left ventricle. Specifically, an acquisition sequence is set up providing count data sets collected at subsequent time intervals. For each data set, an image reconstruction algorithm is applied, ROIs are drawn within the left ventricle, the kidneys, and the bladder, and the corresponding tracer concentrations are computed. Obviously, the tracer concentration in the kidneys is an estimate of  *C*_*t*_ + *C*_*p*_  plus the tracer carried by the blood contained in the kidneys' vascular system. This last term cannot be identified in the images and will be accounted for in the optimization procedure. Specifically, the optimization scheme we will implement minimizes, at each time point, the functional

(25) $$\begin{array}{c} 𝒞∶={||\left(C_{t}+C_{p}\right)-{\bar{C}}_{\exp}||}^{2}+{||C_{u}-{\bar{C}}_{u}||}^{2}, \end{array}$$

where  *C*_*u*_, *C*_*t*_, and *C*_*p*_ are the analytical solutions of the direct problem computed at the given time point; ${\bar{C}}_{u}$ is the concentration measured from the ROI in the bladder at the time point; ${\bar{C}}_{\exp}∶=\left({\bar{C}}_{k}-V_{b}C_{b}\right)/\left(1-V_{b}\right)$, where ${\bar{C}}_{k}$ is the concentration measured from the ROI on the kidneys, *C*_*b*_ is the value of the TAC at the specific time value, and *V*_*b*_ measures the blood fraction with which the kidneys' vascular system is supplied. In the following, we will assume *V*_*b*_ = 0.2, which is a physiologically sound value [13].

The minimization of the functional *𝒞* is realized by means of an Ant Colony Optimization (ACO) scheme [9]. ACO is a statistical-based optimization method developed in the 1990s with the aim of providing a reliable although not optimal solution to some nondeterministic polynomial-time hard combinatorial optimization problems. While an ant is going back to the nest after having taken some food, it releases a pheromone trace that serves as a trail for next ants, which are able to reach food detecting pheromone. Since the pheromone decays in time, its density is higher if the path to food is shorter and more crowded; on the other hand, more pheromone attracts more ants, and at the end, all ants follow the same trail. This behavior is paraphrased in ACO identifying the cost functional *𝒞* with the length of the path to food and the pheromone traces with a probability density which is updated at each iteration depending on the value of the cost function for a set of states. In practice, at each iteration, the cost function is evaluated on a set of *P* admissible states, and the states are ordered according to increasing values of the cost function. Then ACO defines a probability distribution which is more dense in correspondence with the cheaper states, and, on its basis, *Q* new states are extracted. A comparison procedure identifies the new best *P* states which form the next set of states. Formally, the starting point of the algorithm is a set of *P* states

(26) $$\begin{array}{c} B∶=\left\{U_{k}=\left(u_{1,k},\ldots ,u_{N,k}\right)\right\},\; \end{array}$$

such that

(27) $$\begin{array}{c} U_{k}\in S\subset {\mathbb{R}}^{N},\;k=1,\ldots ,P, \end{array}$$

that are ordered in terms of growing cost; namely, *𝒞*(**U**_1_)≤⋯≤*𝒞*(**U**_*P*_). Next, for each *j* = 1,…, *N* and *i* = 1,…, *P*, one computes the parameters

(28) $$\begin{array}{c} m_{i,j}=u_{j,i},\;\;s_{i,j}=\frac{\xi }{P-1}\sum_{p=1}^{P}\left|u_{j,p}-u_{j,i}\right| \end{array}$$

and defines the probability density function

(29) $$\begin{array}{c} {𝒢}_{i}=\sum_{i=1}^{P}w_{i}{𝒩}_{\left[m_{i,j},s_{i,j}\right]}\left(t\right), \end{array}$$

with *i* = 1,…, *P*, *ξ*, *q*, real positive parameters to be fixed, and *w*_*i*_ = *𝒩*_[1,*qP*]_(*i*). By sampling *S* *Q* times with *𝒢*_*j*_, the procedure generates *Q* new states **U**_*P*+1_,…, **U**_*P*+*Q*_, enlarging the set *B* to $\tilde{B}=\{U_{1},\ldots ,U_{P+Q}\}$. If **U**_*k*_1__,…, **U**_*k*_*Q*__ are the *Q* states of $\tilde{B}$ of greater cost, the updated *B* is defined as

(30) $$\begin{array}{c} B=\tilde{B}\setminus \left\{U_{k_{1}},\ldots ,U_{k_{Q}}\right\}. \end{array}$$

This procedure converges to an optimal solution of the problem by exploiting the fact that the presence of the weights *w*_*i*_, in the definition of *𝒢*_*j*_, gives emphasis to solutions of lower costs since *w*_1_ > ⋯>*w*_*P*_. This fact, associated with the influence that a proper choice of parameters *ξ* and *q* has on the shape of the Gaussian functions, determines the way in which the method tunes the impact of the worse and best solutions. The algorithm ends when the difference between any two states of *B* is less than a predefined quantity or when the maximum allowable number of iterations is reached. The initial set *B* of trial states is chosen by sampling a uniform probability distribution.

The implementation of ACO for the optimization of the exchange coefficients in *𝒞* is based on the following steps.

(1) The four ACO parameters are fixed as follows.  *P*  and  *Q*  are chosen as in [14]. Specifically,  *P*  is a multiple of the number of coefficients to optimize plus one and
  (31) $$\begin{array}{c} Q=\left[\frac{P}{2}\right]+1, \end{array}$$
 where [·] indicates the floor and *q* and *ξ* are fixed searching for a trade-off between the risk of a solution space of a too high complexity and the risk of a too high computational demand. In order to realize this trade off, we have applied a heuristic procedure based on the outcome of the experiments with synthetic data (see Section 3.1). Specifically, in all applications, we have used *ξ* = 0.4 and *q* = 10^−2^. Anyhow, the method is very robust with respect to the choice of *ξ* and *q*.
(2) The values of the tracer coefficients are initialized to six random numbers picked up in the interval, respectively,  (0.5,1.5)  for the coefficient  *k*_*bt*_  and  (0,1)  for the others coefficients (this choice is based on the literature [15]).
(3) The ACO procedure is then run using *C*_*u*_, *C*_*t*_, and  *C*_*p*_ as the solutions in the general full matrix case. If, during the ACO iterations, the reconstructed values for the tracer coefficients become statistically consistent with values for which the associated direct problem is described by a triangular or a diagonal matrix, the algorithm automatically switches to utilize the corresponding solution in the computation of  *𝒞*.

In the following section, we show how this statistics-based compartmental analysis works in the case of synthetic data and real measurements recorded by a micro-PET system. In this specific application, the advantages of ACO with respect to deterministic optimization are that it does not suffer local minima and singularities in the functional gradient.

## 3. Results and Discussions

Compartmental analysis is a valid approach to physiological studies of animal models by means of PET data. An “Albira” micro-PET system produced by Carestream Health is currently operational at the IRCCS San Martino IST, Genova, Italy, and experiments with mice are currently performed by using different tracers, mainly for applications to oncology. In this section, we describe the performance of our approach to compartmental analysis in the case of synthetic data simulated by mimicking “Albira” acquisition for FDG-PET experiments. Then, we will describe the results of data analysis for five real experiments performed by using FDG.

### 3.1. Application to Synthetic Data

**Figure 2:**
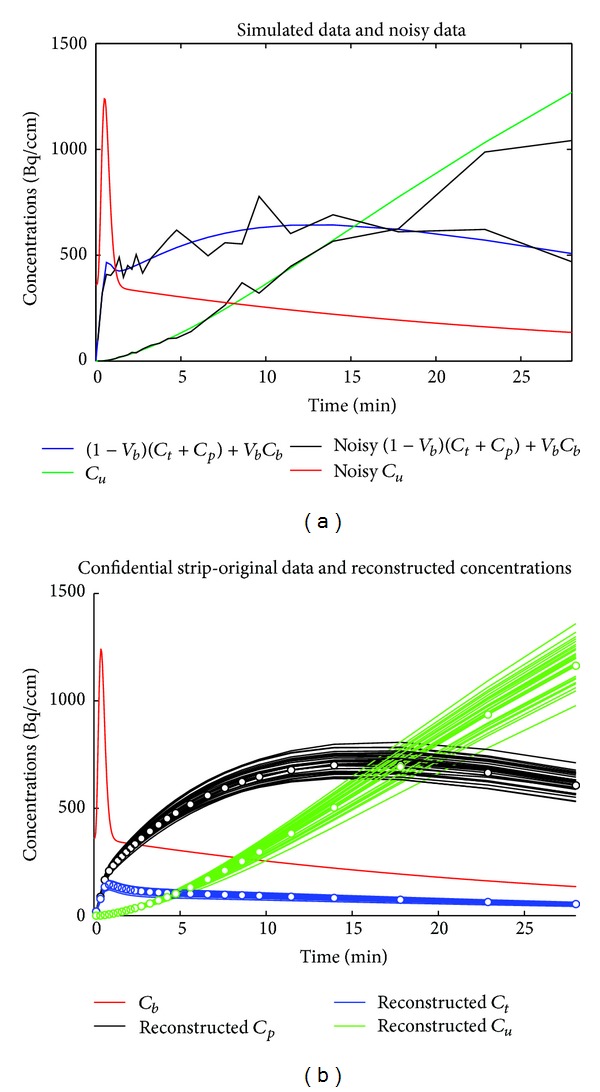
Simulated experiment with a full matrix **A** as in (4). Results obtained with the following ACO parameters: *P* = 13, *Q* = 7, *q* = 0.015, and *ξ* = 0.4 for 30 runs of the algorithm. (a) Red line represents *C*_*b*_, green line represents *C*_*u*_, and blue line represents the total measurement on kidneys. In black, the same data corrupted by Poisson noise. (b) Superimposition of synthetic data (white dots) and reconstructed confidence strips of concentrations.

 In order to produce the synthetic data, we started from six initial values for the tracer coefficients. These selected values generate a matrix  **A**  and a vector  **K**  as in (4) and (5). The corresponding solutions for  *C*_*t*_,  *C*_*p*_, and  *C*_*u*_  associated with the Cauchy problem (1) are given in (8), (9), and (15). The solutions are sampled at 27 time points corresponding to the distribution of acquisition times performed by “Albira” for this kind of experiments. The red line in Figure 2(a) represents the TAC  *C*_*b*_  that has been obtained by fitting with a gamma variate function a set of real measurements acquired from a healthy mouse in a very controlled experiment [16]. The vectors corresponding to the discretization of  *C*_*t*_  and  *C*_*p*_  are then summed together and affected by Poisson noise; the same kind of noise is applied to the vector corresponding to the discretization of  *C*_*u*_, as represented in Figure 2(a). ACO is applied 30 times to these synthetic data set to obtain 30 sets of reconstructed values of the exchange coefficients; in correspondence with each set we then computed the concentration curves and superimposed them in Figure 2(b), thus producing confidence strips for the reconstructed concentrations. The same kind of experiment has been performed again, choosing as initial values the ones that give rise to a lower triangular system as in (16), an upper triangular system matrix as in (20), and a diagonal system as in (23). In Table 1, we present the average values of the tracer coefficients and the standard deviations of the values reconstructed by the 30 runs of ACO over the same set of input vectors. The results show that ACO is reliable in reproducing the ground truth values. In the cases of the upper triangular and diagonal matrices, the reason why, for some coefficients, ACO gives zero to the average and standard deviation is due to the fact that our implementation contains a threshold for the parameter outputs (equal to  10^−3^). In this experiment, these coefficients are underthreshold for all runs, and therefore the averages and standard deviations are set to null.

The main advantage of using ACO for the reduction of this compartmental model is in the fact that this statistical approach, as most evolutionary methods, is particularly effective in exploring the solution space. This property becomes evident by performing the same analysis of synthetic data by means of a standard least-squares method. Therefore, we have utilized the Levenberg-Marquardt (LM) approach [17] for a comparison test. We note that ACO is a statistical method, and, for a given initialization, different runs of ACO produce different results. On the contrary, LM is a deterministic method, and therefore, in our tests, we have applied the method 30 times using 30 different initializations. However, each initialization has been chosen as in ACO, that is, by randomly drawing the six values of the tracer coefficients in the intervals (0,1) for  *k*_*tp*_, *k*_*pt*_, *k*_*up*_, *k*_*tb*_, and *k*_*pb*_  and in  (0.5,1.5)  for  *k*_*bt*_. The results of this test are again in Table 1, reporting the average values and the corresponding standard deviations over the 30 realizations for the same data sets as in the ACO experiment. These results show that ACO is more accurate in reproducing the ground truth values and that it does this with smaller uncertainties.

We agree that in these tests, the procedure for generating the synthetic concentrations and the one for reconstructing the tracer coefficients from them are based on the same equations (in a sort of “inverse crime” procedure). However, the synthetic data are affected by Poisson noise, and in any case, the aim of these numerical applications was simply to validate the reliability and stability of ACO when applied, for the first time, to a compartmental analysis problem.

### 3.2. Application to Real Measurements

**Figure 3:**
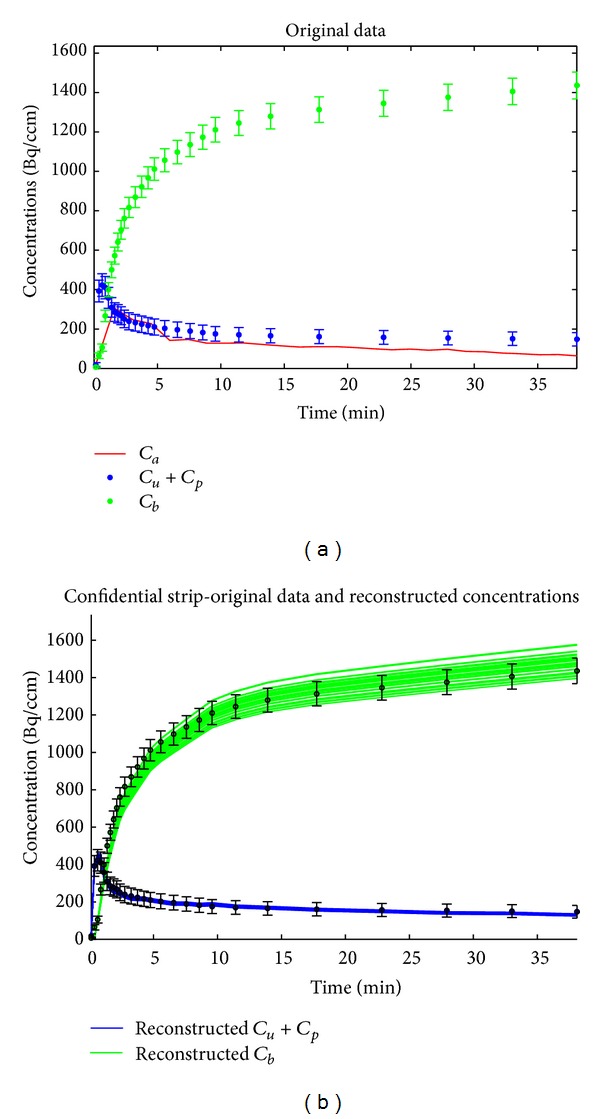
Analysis of real data from one of the murine models. Results obtained with the following ACO parameters: *P* = 25, *Q* = 13, *q* = 0.0001, and *ξ* = 0.65 for 20 runs of ACO. (a) Red line represents *C*_*b*_; blue dots represent *C*_*t*_ + *C*_*p*_ while green dots represent *C*_*u*_. (b) Superimposition of concentrations in the bladder (green) and in the kidneys (blue) computed by solving the forward problem where the tracer coefficients are reconstructed by ACO. The error bars are Poisson that correspond to the square root of the measured counts.

 We considered five healthy murine models injected with FDG and acquired the corresponding activity by means of a dynamic acquisition paradigm over 27 experimental time points. The images have been reconstructed by applying an expectation-maximization iterative algorithm [18], and ROIs have been drawn on the reconstructed images around the left ventricle to reproduce the time activity curve. ROIs have been also drawn around the kidneys and the bladder in order to compute the input concentrations. In Figure 3(a), concerning one of the mice, the red line describes the TAC  *C*_*b*_  (we have plotted the solid line connecting the measured concentrations in order to distinguish the input function from the other concentrations) while the green points represent the concentrations for the bladder. The blue points correspond to the measured concentration in the kidneys, and the error bars correspond to the square root of the measured counts. Then, ACO has been applied 30 times against these data. The initialization values for the tracer coefficients are the same for all 30 runs and are obtained by drawing randomly *k*_*bt*_ in (0.5,1.5) and the others five coefficients within  (0,1). Then, for each run, the set of reconstructed tracer coefficients are used to solve the direct problem in order to obtain reconstructions of  *C*_*u*_  and  *C*_*p*_ + *C*_*t*_. The confidence strips resulting from the superposition of the 30 reconstructions of the concentration are represented in Figure 3(b).

**Figure 4:**
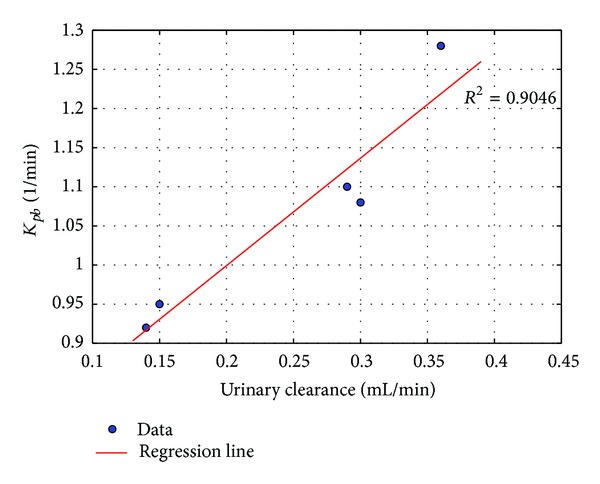
Correlation between the average clearances 〈Cl〉 and rate coefficients *k*_*pb*_ (from blood to preurine) for five healthy models.

The results of this analysis for all models are given in Table 2 containing the average and standard deviation (over the 30 realizations) of the tracer coefficients. These coefficients have dimension minutes^−1^ and can be interpreted as a measure of the effectiveness with which FDG is exchanged from one compartment to another. Table 2 also reports the results obtained by applying LM to the same data sets with 30 different random realizations of the initialization values. The comparison between the performances of the two methods indicates that ACO and LM provide similar average values, although ACO is characterized by smaller uncertainties. Further, these results show that the model we adopted in this analysis can be utilized to quantitatively assess the FDG metabolism in the renal system. For example, the fact that *k*_*pb*_≃*k*_*up*_ and  *k*_*tp*_, *k*_*pt*_  are smaller provides a quantitative evaluation of the excretion of FDG into the bladder once it is transferred into the preurine. Further, *k*_*bt*_ > *k*_*tb*_ shows that the fraction of FDG which is absorbed by the cells (and not directly transferred into preurine) is effectively transported back to the blood flow and therefore again put at disposal of the excretion process. Finally, *k*_*pb*_ can be considered as a quantitative measure of this process, since, as shown in Figure 4, the five *k*_*pb*_ values in Table 2 significantly correlate with the corresponding values of the averaged clearance defined as

(32) $$\begin{array}{c} \langle \text{Cl}\rangle =\frac{C_{u}\left(T\right)V_{u}\left(T\right)}{\int_{0}^{T}C_{b}\left(t\right)dt}, \end{array}$$

where  *T*  is the final acquisition time and  *V*_*u*_  is the volume of the bladder. We observe that this tight correlation between clearance and  *k*_*pb*_  indicates that the two indexes are actually redundant. However, this conclusion would be reasonable for the specific experimental condition of the present study that only included normal mice with (presumably) normal renal function. As for every substance characterized by kidney excretion, renal clearance is the final result of a number of processes starting from the number of molecules available for filtration, filtration itself, possible active excretion in tubule, and (vice-versa) reabsorption. Accordingly, measuring this last parameter by  *k*_*pb*_  allows to identify the degree of substance reabsorption independently from the glomerular filtration rate, whose reduction would inevitably result in reduced FDG clearance. This estimation would be of particular relevance to study the net effect of drugs derived from phloretin aiming to reduce serum glucose levels by reducing tubular sugar reabsorption. More importantly, this method would permit to identify possible pharmacologic interference caused by other drugs on this innovative therapeutic approach to diabetes.

## 4. Conclusions

This paper deals with the renal flow of a radioactive tracer, [^18^F]-FDG, injected into a mouse. The time evolution of tracer concentrations inside kidneys and from kidneys to bladder has been modeled by a linear system of ordinary differential equations with constant coefficients. The time variation of the total concentration of activity inside kidneys and bladder (essentially, the sum of the solutions) has been estimated through an analysis of micro-PET data. The six constant exchange coefficients, which provide information on FDG metabolism, have been regarded as unknowns. The related inverse problem has been solved by applying an algorithm based on ACO. Resulting applications to real and synthetic data have been shown and discussed also in comparison with the results provided by a Levenberg-Marquardt algorithm.

The mathematical approach described in this paper provides estimates of the six unknown coefficients. Unlike techniques based on graphical analysis, it does not require any distinction between irreversible or reversible uptake of tracer nor identification of a time value after which suitable expressions evaluated from the data become linear in time [12]. Moreover, the graphical methods provide fewer parameters, usually slopes and intercepts, which can be interpreted as functions of the original model parameters [19]. Technically, the general character of the optimization procedure based on ACO makes it applicable to compartmental model structures of a variety of types, provided that measured data on the related total concentrations of activity are available. For example, no practical restriction on the number of compartments involved is required if the direct problems involved in the ACO procedure are solved numerically, instead of finding the analytic representation of the solutions, as we have done in the present paper. Similarly, the fully numerical approach does not require limitations such as the constraint that the system matrix is sign-symmetric [20, 21] (indeed, the upper and lower triangular matrices considered in Section 2 do not comply with sign-symmetry). We have also seen that application of ACO to synthetic data corresponding to an upper triangular matrix leads to the final estimate  *k*_*tp*_ = 0. This shows that the ACO approach is also capable of recovering vanishing rate constants. More generally, our computational approach to micro-PET data analysis shows that (1) the tracer coefficients of the compartmental system quantitatively measure the effectiveness of the excretion process; (2) these parameters offer a reliable and more local alternative to clearance, providing quantitative details to a process that clearance is able to describe just in a global way; and, specifically, (3) the average clearance and the rate coefficient from blood to preurine are correlated with a significantly high correlation coefficient.

The physiological basis for this study relies on the broad utilization of FDG in the diagnosing and staging of cancer. In fasting patients, this tracer accurately maps the insulin independent glucose metabolism as an index of aggressiveness and growth rate of neoplastic lesions. We agree that MRglc determined by means of, for example, Patlak analysis would provide a reliable quantitative index of glucose consumption. However, these measurements imply the use of dynamic imaging whose long acquisition time (50–60 minutes) would hardly fit with the operational procedures of a PET lab. Accordingly, clinical PET imaging almost always implies the acquisition of only one image at the late (equilibrium) time. Under this condition, only tracer uptake can be measured. SUV is largely used to define cancer glucose consumption [22] but obviously also depends upon tracer availability. Most often, this latter variable is neglected since blood FDG clearance by tissues is assumed to be relatively stable in the different patients. Nevertheless, differently from glucose, a significant amount of FDG is excreted in the urine. This tracer sequestration has been already documented and attributed to the low affinity of glucose symporters dedicated to glucose reabsorption from preurine in the kidney tubules [4, 23]. Treatments or conditions able to modify FDG availability would inevitably affect SUV values independently from cancer glucose metabolism. Our study aimed to document whether this phenomenon occurs and to what degree it can affect the conventional representation of FDG uptake. As a matter of fact, phloretin-like drugs acting on SGLT2 (one of the two mechanisms devoted to tubular glucose reabsorption) are now entering the market. Considering the pandemic dimension of diabetes, an accurate evaluation of their interference on FDG kinetics would be of great interest to define whether or not SUV remains a reliable marker of tumor metabolism.

## Figures and Tables

**Table 1:** Simulated values of tracer coefficients providing different cases for the matrix **A**; reconstructed average values and standard deviations over 30 runs of ACO (same random initialization guess) and over 30 runs of LM (30 different random initializations). Values under 10^−3^ are set to 0.

	*k*_*bt*_	*k*_*tp*_	*k*_*pt*_	*k*_*up*_	*k*_*tb*_	*k*_*pb*_
**A** full						
g. t.	1	0.02	0.02	0.08	0.3	0.3
ACO	1.01 ± 0.11	0.02 ± 0.01	0.02 ± 0.01	0.08 ± 0.01	0.32 ± 0.03	0.31 ± 0.02
LM	1.13 ± 1.04	0.02 ± 0.06	0.03 ± 0.05	0.08 ± 0.04	0.28 ± 0.29	0.31 ± 0.26
**A** u. t.						
g. t.	0.8	0	0.02	0.1	0.4	0.2
ACO	0.88 ± 0.10	0 ± 0	0.02 ± 0.01	0.11 ± 0.01	0.41 ± 0.05	0.20 ± 0.02
LM	0.92 ± 0.71	0 ± 0	0.06 ± 0.05	0.16 ± 0.22	0.39 ± 0.25	0.19 ± 0.11
**A** l. t.						
g. t.	0.6	0.03	0	0.1	0.35	0.35
ACO	0.59 ± 0.05	0.03 ± 0.01	0 ± 0	0.10 ± 0.01	0.35 ± 0.02	0.35 ± 0.02
LM	0.64 ± 0.33	0.06 ± 0.08	0 ± 0	0.09 ± 0.12	0.36 ± 0.17	0.39 ± 0.21
**A** diag.						
g. t.	0.7	0	0	0.2	0.2	0.4
ACO	0.71 ± 0.03	0 ± 0	0 ± 0	0.21 ± 0.01	0.20 ± 0.01	0.41 ± 0.03
LM	0.74 ± 0.36	0 ± 0	0 ± 0	0.17 ± 0.11	0.25 ± 0.12	0.46 ± 0.31

In the first columns: g. t. stands for ground truth, u. t. for upper triangular, l. t. for lower triangular, and diag. for diagonal.

**Table 2:** Results of the data analysis in the case of 5 murine models. Reconstructed average values and standard deviations over both ACO (30 runs over the same random initialization) and LM (30 different random initializations).

	*k*_bt_	*k*_*tp*_	*k*_*pt*_	*k*_*up*_	*k*_tb_	*k*_*pb*_
1ACO	1.16 ± 0.39	0.03 ± 0.02	0.04 ± 0.03	0.31 ± 0.06	0.22 ± 0.02	0.26 ± 0.02
1LM	1.32 ± 1.64	0.07 ± 0.11	0.05 ± 0.09	0.41 ± 0.62	0.17 ± 0.21	0.29 ± 0.22

2ACO	0.93 ± 0.16	0.07 ± 0.04	0.04 ± 0.02	0.21 ± 0.03	0.19 ± 0.02	0.19 ± 0.03
2LM	1.16 ± 1.12	0.10 ± 0.09	0.04 ± 0.06	0.29 ± 0.31	0.22 ± 0.17	0.20 ± 0.13

3ACO	0.93 ± 0.13	0.04 ± 0.02	0.03 ± 0.01	0.21 ± 0.07	0.19 ± 0.03	0.19 ± 0.04
3LM	0.88 ± 1.01	0.05 ± 0.05	0.04 ± 0.05	0.22 ± 0.19	0.18 ± 0.20	0.19 ± 0.14

4ACO	1.19 ± 0.18	0.06 ± 0.03	0.02 ± 0.01	0.38 ± 0.11	0.32 ± 0.03	0.31 ± 0.03
4LM	1.11 ± 1.41	0.07 ± 0.09	0.02 ± 0.05	0.43 ± 0.39	0.27 ± 0.31	0.30 ± 0.23

5ACO	1.11 ± 0.10	0.06 ± 0.03	0.05 ± 0.03	0.32 ± 0.02	0.33 ± 0.02	0.29 ± 0.02
5LM	1.03 ± 1.31	0.08 ± 0.07	0.05 ± 0.06	0.27 ± 0.29	0.33 ± 0.41	0.27 ± 0.19

In the first columns, 1ACO indicates the results concerning the first murine model provided by ACO and so on.
